# A Comparison of the Physiological Responses to Underwater Arm Cranking and Breath Holding Between Synchronized Swimmers and Breath Holding Untrained Women

**DOI:** 10.2478/v10078-012-0031-7

**Published:** 2012-05-30

**Authors:** Teresa C. Alentejano, Gordon J. Bell, Dru Marshall

**Affiliations:** 1 University of Alberta, Edmonton, Alberta, Canada..; 2 University of Calgary, Calgary, Alberta, Canada.

**Keywords:** water immersion, heart rate, oxygen consumption, ventilation

## Abstract

Exercise and breath holding in the water such as that performed in the sport of synchronized swimming may evoke the physiological consequences of the diving response. The purpose of this study was to investigate the physiological responses of breath holding during underwater arm cranking in synchronized swimmers who are trained in breath holding and compare these responses to untrained women. Each participant performed 6 breath holding periods in the water (2 × 10s, 2 × 20s and 2 × 25s) with 2 minutes of normal breathing in between, in either an ascending or descending order while performing arm crank exercise. The intensity of arm crank exercise was set below the individual ventilatory threshold. Both synchronized swimmers and controls were matched on sitting height and then randomly divided into 2 groups: one group started breath holding with the longest (25s) breath holding periods while the other group began breath holding with the shortest (10s) breath holding periods. The synchronized swimmers experienced a significant decrease in heart rate while breath holding for 20 and 25s but the changes in heart rate for the control group was not consistent between subgroups. Full recovery from breath holding was identified for minute ventilation after 25s of recovery from breath holding for all groups. Results suggest synchronized swimmers exhibited a better adaptation to breath holding while exercising underwater.

## Introduction

Synchronized swimming is a sport that involves complex movements of varying exercise intensity, some of which are performed underwater while breath holding. Research in our lab has shown that during a national competition, soloists immersed their face an average of 18 times and for an average of ∼7 seconds, but the longest breath hold period lasted ∼39 seconds (Alentejano et al., 2008). Furthermore, trained synchronized swimmers exhibited longer breath hold periods with similar physiological responses but at a lower heart rate during recovery in comparison to women not trained in breath holding (Alentejano et al., 2010). Several studies have examined breath holding in synchronized swimmers while exercising, but have been limited to reports of the partial pressure of oxygen and carbon dioxide in relation to safety issues (Davies et al., 1993; Davies et al., 1995); heart rate and lactate responses during the performance of figure exercises (Gemma and Wells, 1987; Figura et al., 1993); physiological responses to simulated synchronized swimming exercise (Bante et al., 2007); and, ventilatory responses measured out of the water (Naranjo et al. 2006). It is also known that synchronized swimmers hold their breath for different lengths of time that vary throughout a routine while exercising underwater (Alentejano et al., 2010). However, the physiological responses to different lengths and order (ie, longest period at the front, middle, or end of a routine) of breath holding while exercising underwater has not been well studied.

The present study was conducted to provide a better understanding of the cardio-respiratory responses of synchronized swimmers while breath holding during exercise at a controlled intensity while immersed in water. As well, a unique aspect to the present study was to examine these responses to different sequences of breath hold durations. Therefore, the purpose of this study was to investigate the effect of a series of breath holding periods of 10, 20 and 25s in ascending or descending order on a variety of cardio-respiratory responses in synchronized swimmers while exercising underwater and whether these differed from BH untrained women.

## Methods

### Subjects

The present study was conducted as a part of a larger research project, part of which has been previously reported by our lab (Alentejano et al., 2010). The participants were 15 women from competitive synchronized swimming clubs (SS) and 15 women who served as the comparison group (C). These latter women were matched on sitting height to the SS women. They were also screened to make sure that they were comfortable holding their breath while immersed in water and that they had not participated in high level of competitive swimming or anything that involved breath holding (BH). Other requirements included that the subjects were non-smokers, had ability to BH for 45 s at rest and had a ratio of forced expiratory volume in 1 s to forced vital capacity at a minimum of 75%. An institutional research ethics board provided approval for this study and all participants were required to provide written consent before participating. Participant characteristics, experience and related demographics have been reported previously (Alentejano et al., 2010).

Briefly, each participant attended two orientation sessions for screening; completed all questionnaires; had their body mass, standing height, sitting height, spirometry and BH ability assessed at rest; were instructed on how to use the apparatus custom designed for underwater exercise using a modified Monark ergometer (Sweden) adapted from Chen et al. (1996); and, were immersed to the clavicular notch in chlorinated water in a small tank and allowed to practice BH while using the experimental mouthpiece apparatus as previously detailed for our laboratory (Alentejano et al., 2010).

### Procedures

Each participant completed a graded exercise test to volitional exhaustion while arm cranking in the water. All participants wore a water resistant heart rate monitor set to record HR every 5 s to memory (Polar Vantage, Kempele, Finland). A headpiece and mouthpiece apparatus was rigged to enable metabolic measurements to be made while exercising in the water as previously detailed for our laboratory (Alentejano et al., 2010). The participants donned a nose clip and were strapped to the seat of the underwater apparatus. They also wore waist and ankle weights to assist with remaining submerged and were allowed to use swim goggles (optional). Again, the water level was adjusted to the clavicular notch and temperature was 28 ± 1°C.

The metabolic measurement system (MedGraphics CPX/D, Minn) was calibrated according to the manufacturers and operated in the breath by breath mode while heart rate was obtained every 5s. After 2 min of non-exercise acclimatization in the water, the participants subjects were instructed to complete 4 to 5 minutes of unloaded arm cranking (except for water resistance) as a warm-up followed by a graded exercise test to volitional exhaustion to determine peak oxygen consumption (VO_2peak_) while immersed in water. Arm cranking rate was set at 60 rpm and controlled with a metronome. Initial power output was set at approximately 40 W (∼29.4 W from the cycle ergometer resistance and ∼11 W from the water resistance as previously determined in our lab) with an additional increment of approximately 15 W every 2 min until volitional exhaustion. The test was terminated when the subject could not continue due to fatigue as evidenced by an inability to maintain an arm crank cadence above 50 rpm. A cool-down period was provided. The ventilatory threshold (VT) was determined as the point where the VO_2_/VCO_2_ ratio reached a minimum value coincident with F_E_CO_2_ reaching a maximum value (Bhambhani and Singh, 1985).

### Breath Hold Trials

**Figure 1:**
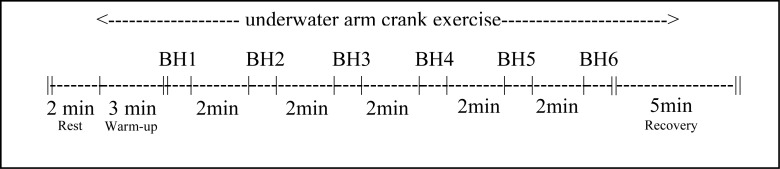
Breath holding (BH) and arm crank exercise experimental procedure

After 3 to 5 days, each participant returned to the laboratory to complete a series of six BH trials of various lengths with the face tilted (immersed) in water while exercising over a period of 16 min and 50 s using the same equipment and setup as previously described. The BH trials started after a 3-min exercise warm-up period with a PO approximately 15 W below the PO where the VT was previously detected. Exercise intensity was maintained from warm-up through to the end of the recovery period for the 6^th^ BH period. Exercise pace was controlled by a metronome set at 60 b·min^−1^. Breath holding intervals were 10, 20, or 25 s in length and were followed by a 2-min active recovery period. Following this, the participants performed a 5-min recovery that involved 2 min of exercise and 3 min of rest (Figure 1). Due to ethical safety concerns, BH was limited to a maximum of 25 s and a certified lifeguard was present for all trials.

The SS and C participants were matched on sitting height and randomly assigned to two experimental groups. The first group performed the 25 s BH first and continued in order of decreasing BH duration (e.g. 2 x 25 s, 2 x 20 s, 2 x 10 s) and were designated, “*SS 25 s initial”* (n = 7) and *“C 25 s initial”* (n = 7). The other group performed the 10 s BH first and continued in order of increasing BH length (2 x 10 s, 2 x 20 s, 2 x 25 s) and were named “*SS 10s initial”* (n = 8) and *“C 10s initial”* (n = 8). Note that for the 20 and 25 s BH maneuvers, the researcher used an inventive “dummy breathing procedure” previously described, in order to ensure continuous data collection on the metabolic cart was attained during these BH durations (Alentejano et al., 2010). The HR monitor and calibrated metabolic measurement system (MedGraphics CPX/D, St. Paul, Min.) were started simultaneously. The heart rate monitor was set to record HR to memory every 5 seconds and the metabolic measurement system was operated in the breath by breath mode.

### Statistical and Data Analysis

A two-way analysis of variance (ANOVA) with repeated measures was used to evaluate any significant changes in the HR, metabolic and respiratory parameters for the BH periods and whether there were any differences between SS and controls. T-tests were used as post hoc to determine where differences occurred. T-tests were also used to compare group physical characteristics. SPSS software program version 7.5 for Windows^®^ was used for all analyses and alpha was preset at p ≤ 0.05.

## Results

Subject characteristics by experimental group are reported in Table 1. The only significant difference between SS and C is that C subjects were significantly older.

There were no significant differences for P_ET_CO_2_ between groups while P_ET_O_2_ was significantly lower for SS when the BH trials were performed in an ascending order (Table 2). The results also indicated that as BH time increased, P_ET_O_2_ decreased significantly for SS and C but no differences were observed for P_ET_CO_2_. P_ET_CO_2_ was significantly higher for SS than for C but not P_ET_O_2_ when the BH trials were performed in descending order. Note that two BH untrained subjects from the “10s initial” group were only able to finish one 25s BH period.

**Figure 2A–C:**
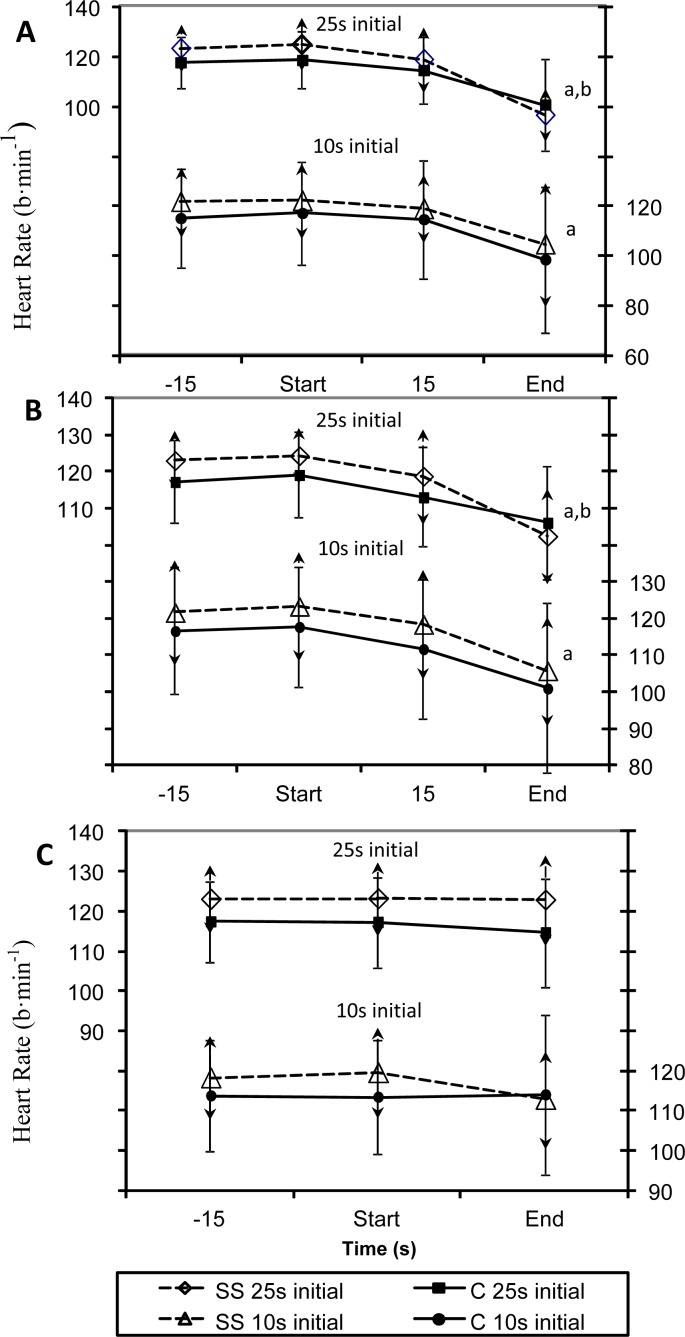
Heart rate responses during breath holding and arm crank exercise in synchronized swimmers (SS 25s initial and SS 10s initial) and controls (C 25s initial and C 10s initial) separated by order in which the breath holding (BH) trials were performed (long BH first vs. short BH first). Values are x̄ ± SD.A. Participants held their breath for 25s. B. Participants held their breath for 20s.C. Participants held their breath for 10s. a = HR is significantly different from the start of BH to the end for the SS group regardless of BH order, p<0.05. *b = HR is significantly different from the start of BH to the end for the C 25s initial group, p<0.05*.

Figure 2 shows that HR response from before to the end of the BH trials for both groups and conditions. There was a significant decrease in HR after the 20 and 25s BH during exercise for the SS group regardless of the order that the BH was performed. However, HR significantly decreased in the C group only when the BH was performed in a descending order.

**Figure 3A–C:**
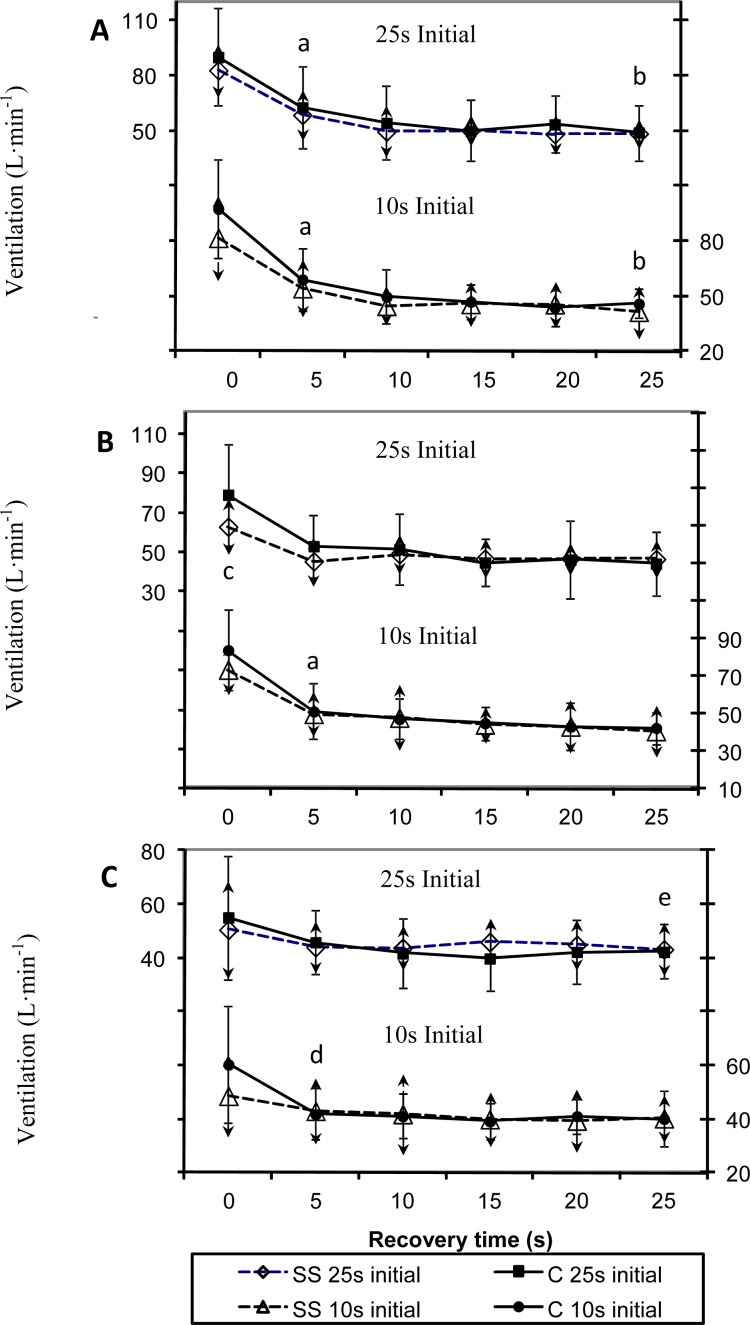
Ventilation ( V_E_) during recovery after breath holding (BH) and arm crank exercise for synchronized swimmers (SS 25s initial and SS 10s initial) and controls (C 25s initial and C 10s initial) separated by order in which the breath holding (BH) trials were performed (long BH first vs. short BH first). Values are x̄ ± SD. A. Participants held their breath for 25s. B. Participants held their breath for 20s. C. Participants held their breath for 10s and a =V_E_is significantly different from the end of BH to 5s into recovery for both SS and controls, p<0.05. b =V_E_is significantly different from 5s into recovery to 25s into recovery, P<0.05. c =V_E_is significantly lower for SS than Controls after 20s of BH when performed in a descending order, p<0.05. d =V_E_is significantly different from the end of BH to 5s into recovery just for controls, p<0.05. *e =V_E_is significantly different from the end of BH to 25s into recovery just for controls, p<0.05*.

The effect of BH and order of BH durations on V_E_ during exercise in the SS and C group is shown in Figure 3. V_E_ was significantly lower for SS compared to C after 20s of BH when the BH trials were performed in a descending order of duration. Furthermore, V_E_ decreased significantly after 5s for both groups when BH for 20s and 25s regardless of the order. There was also significant decrease within 5s for the C group during the 10s BH when performed in an ascending order. As well, V_E_ significant decreased after 25s of recovery for both groups and conditions the 25s BH period

## Discussion

During synchronized swimming competitions, the athletes are required to perform various sequences of movement, many of which are performed underwater. Depending on the routine, the BH maneuvers are performed for different lengths of time with a tendency for the longer BH periods to occur earlier in a soloist’s performance (Alentejano et al., 2008). As a result of this, it was of interest to investigate the physiological responses to BH performed in two sequences. One sequence involved short BH periods performed before longer ones and vice versa. Both trials were conducted while performing steady state arm crank exercise underwater with trained synchronized swimmers and BH untrained women. We found that the end tidal partial pressure of oxygen decreased the longer the breath was held during underwater exercise and was lower in synchronized swimmers compared to controls when the order of BH began with the short BH periods. As well, the end tidal partial pressure of carbon dioxide was higher for synchronized swimmers when the BH periods were performed in the sequence of long to short duration. Heart rate decreased more consistently for SS compared to C during BH. Finally, V_E_ decreased rapidly during recovery from the longer BH times regardless of the order and the synchronized swimmers recovered faster after 20 seconds of BH compared to controls when BH was performed in a descending order of time. Thus, some of the differences observed in the present study would suggest that the synchronized swimmers were less affected and recovered quicker from BH and exercise compared to BH untrained women. As well, there were some differences in the physiological responses to BH and exercise that were dependent on whether longer BH periods were performed before shorter ones or vice versa.

There was an association between the decrease in P_ET_O_2_ and the increase in BH times but not for P_ET_CO_2_. The possible reasons for these differences in gas exchange behavior is that at rest, it has been shown that CO_2_ levels increase rapidly during the first 30s of BH and level off while O_2_ levels keep decreasing until P_A_O^2^ and P_a_O^2^ are similar. (Hong et al., 1971). If the rapid increase of CO_2_ is within 30s of BH at rest, it is possible that during exercise the oxygen exchange rate is increased and the time frame for CO_2_ to level off is decreased. The present study also found a difference in P_ET_CO_2_ between SS and C when the series of BH periods began with the longest breath hold (25 s). In this case SS reached a much higher P_ET_CO_2_ and as the length of BH periods decreased, the P_ET_CO_2_ also decreased for the SS while the P_ET_CO_2_ for group C did not change. This latter finding was independent of the length of the BH maneuver. This is a difficult finding to explain, but this difference may be the result of some training adaptation in the SS. For example, the trained swimmers may have been more efficient, relying on more aerobic metabolic energy production and less on anaerobic glycolysis while BH in comparison to the BH untrained women. However, there are conflicting results about lactate accumulation during BH. Ferreti et al. (1991) reported no lactate increase following BH out of the water despite high oxygen desaturation and Naranjo et al. (2006) reported no changes in lactate during 4 min of exercise and 15 s of intermittent BH for synchronized swimmers. Conversely, Ferrrigno et al. (1997) and Andersson et al. (2004) reported an increase in blood lactate concentration following BH for deep divers and active BH divers performing steady state exercise respectively. Certainly, this conflict deserves further investigation.

Heart rate for the SS decreased significantly during the longer BH periods. Note that there were no changes in HR response after 10 s of BH in either group. The HR decrease for C was slower and only achieved significance when the longer BH trials were performed first. Decreases in HR during exercise and prolonged BH for BH trained subjects have been reported in the literature (Hoffmann et al., 2005; Breskovic et al., 2011; Gemma and Wells, 1987). Our findings are similar to Delapille et al., (2001) who reported higher HR values during exercise with apnea for non BH trained subjects compared to BH trained subjects. The greater HR decrease for the trained swimmers would support a greater adaptation to breath holding in the trained swimmers and this would likely translate into a conservation effect of O_2_.

Minute ventilation increases after BH primarily to provide adequate oxygen for recovery. After 10s of BH, SS did not show need for extra recovery oxygen, since V_E_ remained at a steady state while C showed a significant decrease either within 5s or 25s into recovery. When BH for 25s all groups had a significant drop in V_E_ within 5 s from the start of recovery and a subsequent decrease from 5s to 25s into recovery. Following the 20 s BH period, the synchronized swimmers who performed the longest BH trials first had a significantly lower ventilatory response compared to the untrained group, as shown in Figure 3, which is in accordance with Bjurstrom and Shoene (1987). The difference between the trained and untrained participants at the start of recovery for all the other comparisons did not reach statistical significance even though the general response was similar. The differences observed in the V_E_ between groups could have been the result of a blunted chemoreceptor response in the synchronized swimmers due to the regular exposure to hypoxic conditions while training. Masuda et al. (1981) demonstrated a lower hypoxic sensitivity of the Ama divers associated with an attenuation of the ventilatory response. The lower hypoxic sensitivity was hypothesized to be due to a decreased peripheral chemosensitivity function. From these observations of the ventilatory responses to CO_2_, it seems clear that BH trained subjects perform differently. According to Sanchez and Sebert (1983) when higher levels of CO_2_ are present, there should be an increase in V_E_ response. This did not hold true for the synchronized swimmers compared to the untrained group in the present study. Even though the synchronized swimmers had significantly higher P_ET_CO_2_ values when the longer BH was performed first, there were significantly higher ventilatory responses in the untrained participants following 20s of BH when the longer BH trials were performed first. Sánchez and Sébert (1983) concluded that V_E_ is three times higher during maximal BH before steady state was achieved and recovery of V_E_ from this was faster in sedentary non BH trained subjects. They also observed a decrease in ventilatory response with subsequent BH periods. Furthermore, Naranjo et al (2006) reported V_E_ increases in synchronized swimmers when performing 4 min of exercise with 15 of intermittent BH periods out of the water. In the present study, V_E_ did not significantly vary between the start and the end of exercise. These findings are contrary to previous reports, but BH periods in the present study started after steady state exercise was achieved, which may have made a difference. Ventilation decreased sharply within 5s following BH, achieving steady state in most cases. For longer BH periods of 25s, longer recovery periods were needed. And V_E_ in those cases significantly decreased further from 5 to 25s following BH.

## Conclusion

It is clear that there is a training adaptation to BH while exercising. Breath hold trained subjects such as synchronized swimmers have an advantage versus subjects who are not trained in BH In the present study, breath hold trained subjects significantly reduced their HR during 25s and 20s of BH regardless of what sequence of breath hold length was performed while the decrease in HR was significant only for one of the control subgroups (“25s initial”) when BH for 20 and 25s was performed. Minute ventilation increases following BH were less pronounced in some cases for SS. Further research to determine if there are differences in metabolic energy provision during BH periods dependent on the level of BH training is needed.

As a practical application we would suggest that coaches and choreographers do not need to be concerned about having the longer BH periods at the end of a routine in synchronized swimming competition. In previous research Alentejano et al. (2008) reported that most of the largest BH periods occurred at the beginning of the routines. It was not clear whether this was intentional, ie, it may be that some coaches/choreographers thought that a buildup of fatigue that occurs during a performance may necessitate shorter BH maneuvers near the end of the routine. However, according to the present data, it seems that physiologically there is no difference in having the larger BH periods at the beginning or at the end of the routine, as long as a proper warm up is provided.

## Figures and Tables

**Table 1:** Characteristics for synchronized swimmers (SS 25s initial and SS 10s initial) and controls (C 25s initial and C 10s initial) separated by order in which the breath holding (BH) trials were performed (long BH initially vs. short BH initially). Values are x̄ ± SD

	*SS 25 s initial*	*SS 10 s initial*	*C 25 s initial*	*C 10 s initial*
Age (years)	18 ± 2^a^	18 ± 2^a^	23 ± 2	21 ± 2
Height (cm)	169.9 ± 7.0	171.2 ± 3.2	165.1 ± 7.7	172.7 ± 8.7
Body mass (kg)	58.0 ± 7.3	62.6 ± 6.1	66.56 ± 11.9	65.3 ± 7.1
Sitting height (cm)	88.3 ± 4.1	91.1 ± 1.8	88.7 ± 3.8	91.4 ± 2.2
FVC (L)	4.3 ± 0.8	4.5 ± 0.7	4.0 ± 06	4.2 ± 0.4
Arm Crank VO_2_ peak (L·min^−1^)	1.9 ± 0.4	2.0 ± 0.4	2.2 ± 0.3	2.1 ± 0.4
Arm Crank VO_2_ peak (ml·kg^−1^·min^−1^)	33.0 ± 6.0	31.6 ± 5.6	34.2 ± 5.3	32.0 ± 6.2

*a = significantly different between C “25 s initial” and C “10 s initial” groups, p ≤ 0.05*.

**Table 2:** P_ET_O_2_and P_ET_CO_2_during 6 consecutive breath holding (BH) periods performed in different orders (long BH initially vs. short BH initially) during arm cranking exercise performed underwater for synchronized swimmers (SS 25s initial and SS 10s initial) and controls (C 25s initial and C 10s initial). Values are x̄ ± SD (range)

	*SS 10 s initial*		*C 10 s initial*			*SS 25 s initial*		*C 25 s initial*	

Order of BH	P_ET_O_2_	P_ET_CO_2_	P_ET_O_2_	P_ET_CO_2_	Order of BH	P_ET_O_2_	P_ET_CO_2_	P_ET_O_2_	P_ET_CO_2_
**BH1–10s**	79.6 10.3^c^ (61 – 91)	47.6 4.5 (41 – 53)	83.3 6.9 (74 – 90)	48.4 3.2 (44 – 52)	**BH 1–25s**	65.0 11.5 (47 – 84)	51.9 5.5^c^ (40 – 58)	73.5 10.9 (52 – 89)	44.7 7.6 (36 – 54)
**BH2–10s**	78.3 10.7^c^ (60 – 95)	47.7 3.3 (44 – 52)	84.0 5.5 (75 – 90)	47.4 3.0 (43 – 52)	**BH 2–25s**	64.1 8.1 (48 – 72)	52.5 3.3^c^ (48 – 58)	70.5 18.5 (51 – 92)	45.7 9.8 (30 – 55)
**BH3–20s**	65.3 8.9^a,c^ (51 – 77)	51.4 4.0 (47 – 57)	74.6 10.5^a^ (59 – 89)	46.3 4.3 (41 – 52)	**BH 3–20s**	70.0 7.4^a^ (54 – 79)	50.4 3.4^c^ (45 – 56)	77.0 13.7^a^ (59 – 95)	43.2 6.9 (35 – 50)
**BH4–20s**	68.3 12.3^a,c^ (41 – 84)	49.0 4.3 (46 – 57)	74.8 12.5^a^ (60 – 95)	46.9 6.8 (34 – 53)	**BH 4–20s**	68.7 7.5^a^ (53 – 79)	49.9 3.7^c^ (44 – 56)	72.4 15.0^a^ (59 – 95)	45.7 6.4 (33 – 51)
**BH5–25s**	63.6 17.0^b,c^ (45 – 94)	51.1 7.0 (38 – 59)	68.7 11.1^b^ (54 – 83)	48.6 4.3 (43 – 53)	**BH 5–10s**	83.2 7.2^b^ (66 – 89)	45.6 4.0^c^ (41 – 53)	84.7 8.1^b^ (75 – 96)	44.1 2.6 (39 – 47)
**BH6–25s**	65.3 16.5^b,c^ (45 – 93)	50.8 7.1 (38 – 58)	68.0 12.6^b^ (50 – 82)	48.4 5.5 (41 – 55)	**BH 6–10s**	85.1 7.2^b^ (71 – 96)	44.5 4.3^c^ (38 – 52)	84.5 9.0^b^ (72 – 97)	43.5 3.0 (39 – 48)

*a = significantly different from BH1 and 2, p ≤ 0.05*.

*b = significantly different from BH3 and 4, p ≤ 0.05*.

*c = significantly different from controls, p ≤ 0.05*.

*Note That 2 control participants from the C 10 s initial group were only able to complete 1× 25s BH*.
